# Analysis of the A-B Neuropsychological Assessment Schedule as a Cognitive Screener for Long COVID

**DOI:** 10.7759/cureus.82311

**Published:** 2025-04-15

**Authors:** Kishen Radhakrishna, Jessica Holland, Fiadhnait O'Keeffe, Keith Gaynor, Justin Kinsella, Jessica Bramham

**Affiliations:** 1 Neurology, St. Vincent’s University Hospital, Dublin, IRL; 2 Neuropsychology, St. Vincent's University Hospital, Dublin, IRL; 3 Neurology, St. Vincent's University Hospital, Dublin, IRL

**Keywords:** cognitive neuroscience, covid-19, long covid syndrome, neuropsychology assessment, post-covid-19

## Abstract

Aim

To determine the sensitivity and specificity of the psychometric measures of the A-B Neuropsychological Assessment Schedule (ABNAS) to aid screening of long COVID (LC).

Methods

The participants (N=235) were recruited from an online study of cognitive and psychological consequences of LC, involving individuals attending an LC service in an acute tertiary university hospital and a comparison sample of community controls.The ABNAS for LC, a patient-perceived assessment scale in relation to the challenges they had encountered from LC, was used to identify the specific psychometric measures implicated in LC.

Results

The optimal cut-off value for total ABNAS scores and its psychometric subsets were obtained from receiver operating characteristic (ROC) curves. The sensitivity of the total ABNAS score of ≥21.5 was 81.6% for LC, taken as a post-COVID functional status (PCFS)grade of ≥ 2 as true positives, with a specificity = 72.3%.

The specificity of the ABNAS fatigue subscale score of ≥ 8.5 for LC was 87.2%, while its sensitivity was 66.7%. The sensitivity of the ABNAS mental slowing subscale score of ≥ 4.5 was 82.8%, and the specificity was 70.3%.

Conclusion

Total ABNAS scores and their psychometric subsets (fatigue and mental slowing) are sensitive and specific for LC.

## Introduction

Long-term sequelae of COVID-19 infection, often referred to as "long COVID (LC)," may result in symptoms such as chronic fatigue, "brain fog" or mental slowing, depression, anxiety, cognitive dysfunction and/or worsening of pre-existing co-morbidities which cannot be attributed to another diagnosis [1]. The neuropsychological symptoms of LC may affect daily functioning to variable extents, especially if symptoms persist for over 12 weeks after the acute phase of infection [1]. LC may represent a significant burden to public services by reducing workforce productivity and may affect the quality of life of patients due to its associated cognitive and affective symptoms [1].

Studies have estimated that at least 65 million people globally live with LC, but the actual incidence is thought to be much higher due to undocumented cases [2]. In an Irish context, "post-COVID-19 clinics" should offer multidisciplinary assessments to accommodate the multi-faceted rehabilitation needs of patients with LC [3]. Appropriate neuropsychological assessments at discharge, such as the A-B Neuropsychological Assessment Schedule (ABNAS), could help to streamline rehabilitation referrals and save costs incurred by the Health Service Executive (HSE), Ireland [3,4]. In efforts to improve the assessment processes and provide the best auxiliary services to this vulnerable cohort, it is useful for clinicians to have an assessment tool for identifying LC-related symptoms. The typically used screening tools, such as the Montreal Cognitive Assessment (MoCA), are not particularly sensitive to LC, with an accuracy of 63.3% at detecting any level of impaired neuropsychological performance and an accuracy of 73.3% at identifying extremely low deficits [5]. The objective of this study was to determine the sensitivity and specificity of ABNAS as a novel psychometric measurement tool to aid the screening and management of LC [4].

## Materials and methods

This is a cross-sectional cohort study where a total of 235 participants were recruited from an online study of cognitive and psychological consequences of LC, involving a total of 73 individuals who fulfilled the WHO diagnostic criteria for LC [1], attending a LC service in an acute tertiary referral university hospital that were compared with 162 community controls infected with COVID-19 between March 2022 and February 2023 [6]. The inclusion criteria are participants with self-reported positive tests for COVID-19 at the time of infection, with most participants tested at home, using polymerase chain reaction (PCR) or antigen testing kits. The exclusion criteria applied include incomplete questionnaire responses, pre-existing neurological conditions or prior head injury, and participants aged above 65 to mitigate the possibility of natural degenerative processes on perceived cognitive function. Ethical approval for the study was obtained from the Research Ethics Committee (REC), St. Vincent’s University Hospital, Dublin (REC reference number RS21-065, dated February 22, 2022).

The 73 participants attended the LC clinic for various complaints related to LC symptomatology from March 2022 to February 2023. Of the individuals in the "long COVID group," six had moderate COVID-19 infection (conventional hospitalization without mechanical ventilation) in the acute phase, while four developed severe symptoms requiring mechanical ventilation. This is consistent with published literature that most LC cases are seen in non-hospitalized patients with mild acute illness as this population represented the majority of patients [7].

For the community control sample, the 162 individuals were recruited via an online survey advertised by University College Dublin (UCD), REC reference number RS21-065, open to anyone interested in taking part in the study. All participants completed an online survey that lasted 20 minutes on average. Additional demographic background questions asked included background pre-COVID mental health history. The questions were posed as "Did you experience any psychological difficulties before contracting COVID-19?", with participants given the option to tick "yes" for pre-existing anxiety and/or depression. Participants were given an option to complete the survey at a later time in an effort to reduce the effects of fatigue on questionnaire responses. Participants who had a history of traumatic brain injury or intellectual disability were excluded. The Hospital Anxiety and Depression Scale (HADS) was used in this study to assess the severity of current symptoms of anxiety and depression after contracting COVID-19. 

The presence of LC or "true positives" was taken as patients with at least post-COVID functional status (PCFS) grade 2 (slight functional limitations). The PCFS is a self-reported instrument designed to evaluate the consequences of COVID-19 on functional status [8]. The impact on usual daily tasks is assessed in five scale grades, from grade 0 (no functional limitations) to grade 4 (severe functional limitations) [8].

ABNAS is a patient-perceived psychometric measurement scale, initially introduced as a reliable screening tool for the detection of cognitive deficits associated with epilepsy and anti-seizure medications [4]. It includes 24 items relating to challenges faced in everyday life [4]. Each item is scored on a scale from 0 to 3: a score of 0 indicates no difficulty, 1 mild difficulty, 2 moderate difficulty, and 3 severe difficulty. Cognitive symptoms are evaluated on six domains: fatigue (5 questions), mental slowing (5), memory (4), concentration (4), motor coordination (3), and language (3). The total score ranges from 0 to 72, with higher scores reflecting greater levels of self-reported impairment [9]. ABNAS for LC was selected as a psychometric measurement tool because it had previously demonstrated excellent reliability (Cronbach's α = 0.96) in other studies [4].

The ABNAS for LC, which is a patient-perceived assessment scale in relation to the challenges they had encountered from LC, was used in this study to identify the specific psychometric measures implicated in LC [4]. The sensitivity and specificity of the ABNAS subscales, including fatigue and mental slowing for LC, were also assessed. In this study, the Cronbach's α for the total ABNAS scores and its subsets was 0.814, indicating internal reliability of the scale used for LC (Table 1).

**Table 1:** Reliability statistics

Cronbach’s alpha (α)	Cronbach’s alpha (α), based on standardized items
0.814	0.973

The optimal cut-off values, identified from the Youden’s Index, for the sensitivity of the total ABNAS scores and each of the ABNAS psychometric subscales were determined from receiver operating characteristic (ROC) curves. Sensitivity and specificity tables of the total ABNAS score and relevant psychometric subscales were subsequently constructed. Cronbach's α for total ABNAS scores and its subscales were performed to determine the internal reliability of the scale used.

## Results

The total number of participants from the LC group with depression, regardless of severity, increased to 45 (61.6%) after being infected with COVID-19, n=12 had severe depression, as outlined in Table 2. Among the CC group, n=54 (33.3%) participants reported anxiety pre-COVID. A significant increase in participants with anxiety, regardless of severity, was seen in the CC group after COVID-19, where n=21 cases were severe. In the LC group, 63 (86.3%) patients had PCFS of at least Grade 2.

**Table 2:** Descriptive statistics of all samples, N = 235 LC: long COVID group, CC: community control, PCFS: post-COVID functional status, HADS: hospital anxiety and depression scale

	Group	Number, N (%)	Median	Standard deviation
Number, N	LC	73 (31.1%)	-	-
	CC	162 (68.9%)	-	-
Age	LC	-	48.53	10.21
	CC	-	21	12.85
Gender	LC	Males: 18 (24.7%)	-	-
		Females: 55 (75.3%)	-	-
	CC	Males: 44 (27.2%)	-	-
		Females: 118 (72.8%)	-	-
Hospitalization	LC	9 (4 required mechanical ventilation)	-	-
Pre-COVID mental health history	LC	Depression: n=16 (21.9%)	-	-
		Anxiety: n = 51 (69.9%)		
	CC	Depression: n = 27 (16.7%)		
		Anxiety: n=54 (33.3%)		
Severity of current symptoms of depression and anxiety based on HADS after contracting COVID-19	LC	Total depression post-COVID: 45 (61.6%)	-	-
		Mild: n= 16		
		Moderate: n= 17		
		Severe: n= 12		
		Total anxiety post-COVID: 44 (60.3%)		
		Mild: n= 10		
		Moderate: n=20		
		Severe: n= 14		
	CC	Total depression post-COVID: 33 (20.4%)	-	-
		Mild: n= 25		
		Moderate: n=7		
		Severe: n=1		
		Total anxiety post-COVID: 95 (58.6%)		
		Mild: n= 47		
		Moderate: n = 27		
		Severe: n= 21		
PCFS grade	LC	Grade 0: 7 (9.6%)	-	-
		Grade 1: 3 (4.1%)		
		Grade 2: 24 (32.9%)		
		Grade 3: 34 (46.6%)		
		Grade 4: 5 (6.8%)		
	CC	Grade 0: 90 (55.6%)	-	-
		Grade 1: 48 (29.6%)		
		Grade 2: 17 (10.5%)		
		Grade 3: 4 (2.5%)		

An optimal cut-off value for the total ABNAS score of 21.5 was obtained from the Youden’s Index (0.551) of the ROC curve, area under the curve (AUC) = 0.836 (Appendix A). Using this cut-off value, the sensitivity of a total ABNAS score of at least 21.5 was 81.6% to screen for individuals with LC, taken as a PCFS grade of at least 2 (Table 3). Specificity was 72.3%. 

**Table 3:** Sensitivity and specificity analysis of total ABNAS scores (optimal cut-off of 21.5), derived from Youden’s Index (0.551) of the ROC curve, AUC = 0.836 ABNAS: A-B Neuropsychological Assessment Schedule for long COVID, PCFS: post-COVID functional status, ROC curve: receiver operating characteristic curve, AUC: area under the curve

	PCFS grade 2,3,4 (positives)	PCFS grade 0,1 (negatives)		
Total ABNAS score more than or equal to 21.5	71 (true positives)	41 (false positives)	Positive predictive value 63.4%	Total positives 112
Total ABNAS score < 21.5	16 (false negatives)	107 (true negatives)	Negative predictive value 87%	Total negatives 123
	Sensitivity 81.6%	Specificity 72.3%		Total = 235

An ABNAS fatigue subscale score of at least 8.5 was used as a cut-off value, Youden’s Index (0.538) of the ROC curve, AUC = 0.839 (Appendix B)**.** The specificity of using the ABNAS fatigue subscale score of at least 8.5 to screen for LC was 87.2%, while its sensitivity was 66.7% (Table 4).

**Table 4:** Sensitivity and specificity analysis of ABNAS fatigue subset scores (optimal cut-off of 8.5), derived from Youden’s Index (0.538) of the ROC curve, AUC = 0.839 ABNAS fatigue: A-B Neuropsychological Assessment Schedule fatigue subset score for long COVID, PCFS: post-COVID functional status, ROC curve: receiver operating characteristic curve, AUC: area under the curve

	PCFS grade 2,3,4 (positives)	PCFS grade 0,1 (negatives)		
ABNAS fatigue more than or equal to 8.5	58 (true positives)	19 (false positives)	Positive predictive value 75.3%	Total positives 77
ABNAS fatigue < 8.5	29 (false negatives)	129 (true negatives)	Negative predictive value 81.6%	Total negatives 158
	Sensitivity 66.7%	Specificity 87.2%		Total = 235

An ABNAS mental slowing subscale score of at least 4.5 was used as a cut-off value, Youden’s Index (0.542) of the ROC curve, AUC = 0.838 (Appendix C). The sensitivity of using the ABNAS mental slowing subscale score of at least 4.5 to screen for LC was 82.8%, while its specificity was 70.3% (Table 5).

**Table 5:** Sensitivity and specificity analysis of ABNAS mental slowing subset scores (optimal cut-off of 4.5), derived from Youden’s Index (0.542) of the ROC curve, AUC = 0.838 ABNAS mental slowing: A-B Neuropsychological Assessment Schedule mental slowing subset score for long COVID, PCFS: post-COVID functional status, ROC curve: receiver operating characteristic curve, AUC: area under the curve

	PCFS grade 2,3,4 (positives)	PCFS grade 0,1 (negatives)		
ABNAS mental slowing more than equal to 4.5	72 (true positives)	44 (false positives)	Positive predictive value 62.1%	Total positives 116
ABNAS mental slowing < 4.5	15 (false negatives)	104 (true negatives)	Negative predictive value 87.4%	Total negatives 119
	Sensitivity 82.8%	Specificity 70.3%		Total = 235

The other ABNAS psychometric measures, such as memory, concentration, motor function, and language, produced ROC curves with AUC <0.7; hence, optimal cut-off values were not obtained to determine sensitivity and specificity.

## Discussion

This study revealed that the ABNAS is a sensitive and specific psychometric tool for LC. Total ABNAS scores of at least 21.5 conferred 81.6% sensitivity and 72.3% specificity for LC, taken as a PCFS grade of at least 2. A total ABNAS score of less than 21.5 has a high negative predictive value for LC, which may aid in excluding LC as well. Cronbach's α for the total ABNAS scores and its subsets was 0.814, supporting the reliability of this tool for screening of LC in both clinical practice and trials.

The ABNAS fatigue subscale was found to be a specific measure of LC, revealing a specificity of 87.2% for scores of at least 8.5 but low sensitivity of 66.7%. ABNAS mental slowing scores of at least 4.5 demonstrated 82.8% sensitivity and 70.3% specificity for LC, which may prove to be useful in screening for "brain fog" in patients with LC.

Recent studies have suggested that LC-derived "brain fog" may be attributable to sustained inflammation and disruption of the blood-brain barrier in multiple neuroanatomical regions, including the temporal lobes and frontal cortices [10]. Hence, clinicians are advised to screen for the neurological sequelae of LC, namely cognitive impairment, reduced attention, mental slowing, fatigue, anosmia, and headaches. Early neuropsychological assessment and interventions can be suggested following appropriate screening to improve quality of life while further research for treatment is underway. However, typically used screening tools such as the MoCA are not very sensitive to LC, which is why the ABNAS may be important to uniquely identify the psychometric properties implicated in LC, such as fatigue and mental slowing.

The limitations of this study include a small sample size of 235 patients, consisting of 73 patients in the "long COVID clinical group" and 162 community controls. Hence, further studies should aim to enlist a larger sample to establish generalisability for patients with LC. Secondly, the psychometric measures used in this study, such as the ABNAS and PCFS, are self-reported/patient-perceived limitations to daily function or cognition as opposed to objective measures of cognitive ability. These subjective measures may be implicated by individual perceptions of functioning, mood, or fatigue, which may influence the consistency of the results.

The implementation of a standardized and quick screening method such as the ABNAS is of potentially significant clinical value in LC, where cognitive symptoms such as mental slowing and fatigue are prevalent. Given the increasing burden on neurology and neuropsychology services, a structured screening approach can facilitate the early identification of patients requiring further neuropsychological evaluation, thereby optimizing consultation efficiency and resource allocation. By reducing the time required for initial cognitive screening, this approach enables more rapid and precise triaging of patients to appropriate neuropsychological assessment and intervention pathways. Based on a survey in an Irish cohort, LC had reportedly resulted in a significant impact on the quality of life, ability to perform at work and may cause significant disability [11]. Hence, integrating ABNAS into routine clinical practice has the potential to enhance diagnostic precision, expedite access to targeted cognitive rehabilitation strategies, and ultimately improve patient outcomes in post-COVID cognitive care. Early access to non-pharmacological treatment strategies such as cognitive and physical pacing for chronic fatigue and cognitive impairment have resulted in positive functional outcomes [12,13].

In the United Kingdom, LC has imposed a substantial economic burden [14], underscoring the urgent need for a coordinated, international, and interdisciplinary response [15]. Advancing our understanding and management of this condition necessitates a concerted effort from clinicians, researchers, and policymakers. A multidisciplinary approach, integrating neurology, immunology, rehabilitation medicine, and public health, is essential to mitigate its long-term socioeconomic and healthcare impacts.

## Conclusions

In conclusion, these results indicate that ABNAS has adequate specificity and sensitivity to screen for cognitive symptoms of LC, predominantly fatigue and mental slowing, and is appropriate for use in primary care, neurology consultations, and specialist LC clinics. The ABNAS may be an efficient screening tool in congested clinic services due to the short time required for its completion. Deep consideration should be given to using ABNAS as a novel psychometric measurement to aid early diagnosis and management of LC as the condition requires accurate assessment so appropriate supports may be provided to improve quality of life.

## Figures and Tables

**Figure 1:**
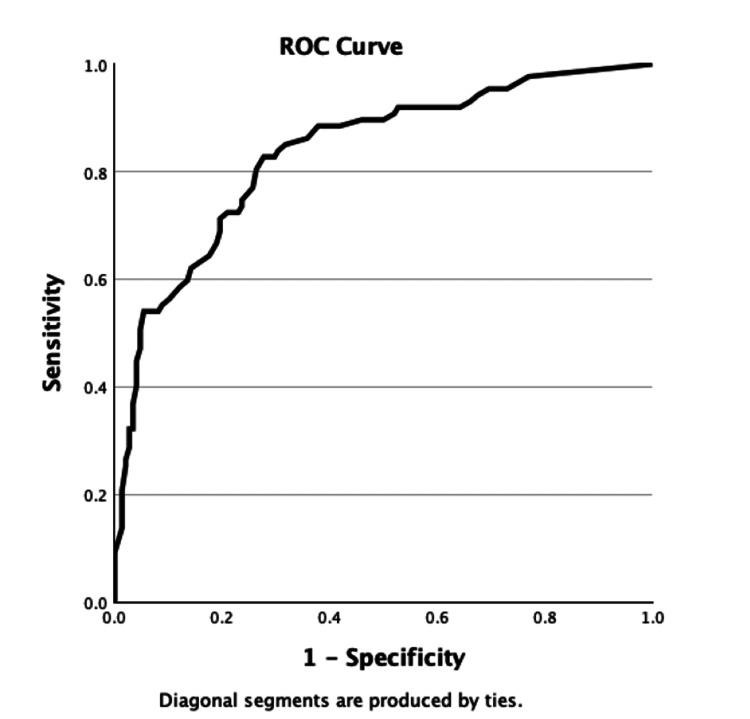
Receiver operating characteristic (ROC) curve of total ABNAS scores against PCFS grades, where grade of at least 2 taken as true positives ABNAS: A-B Neuropsychological Assessment Schedule, PCFS: post-COVID functional status

**Figure 2:**
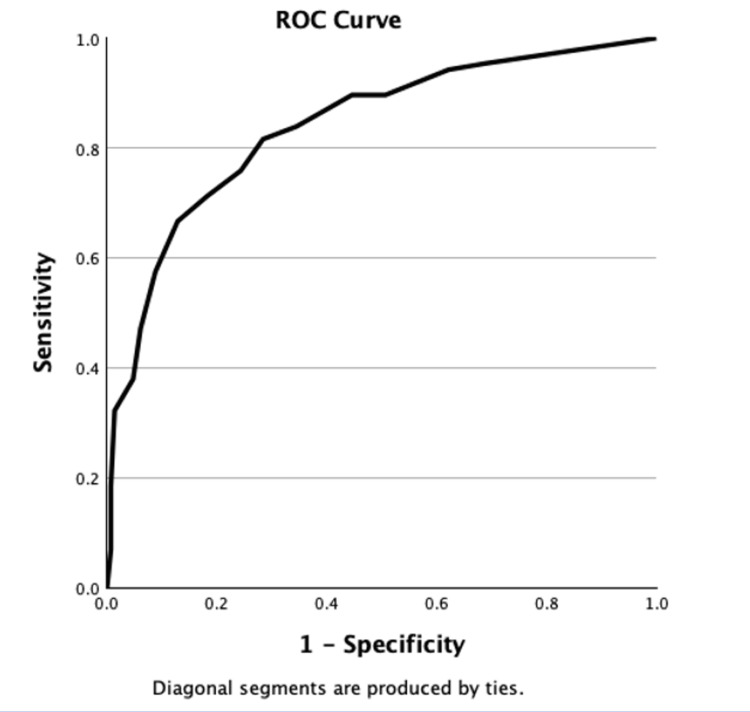
Receiver operating characteristic (ROC) curve of ABNAS fatigue scores against PCFS grades, where grade at least 2 taken as true positives ABNAS: A-B Neuropsychological Assessment Schedule, PCFS: post-COVID functional status

**Figure 3:**
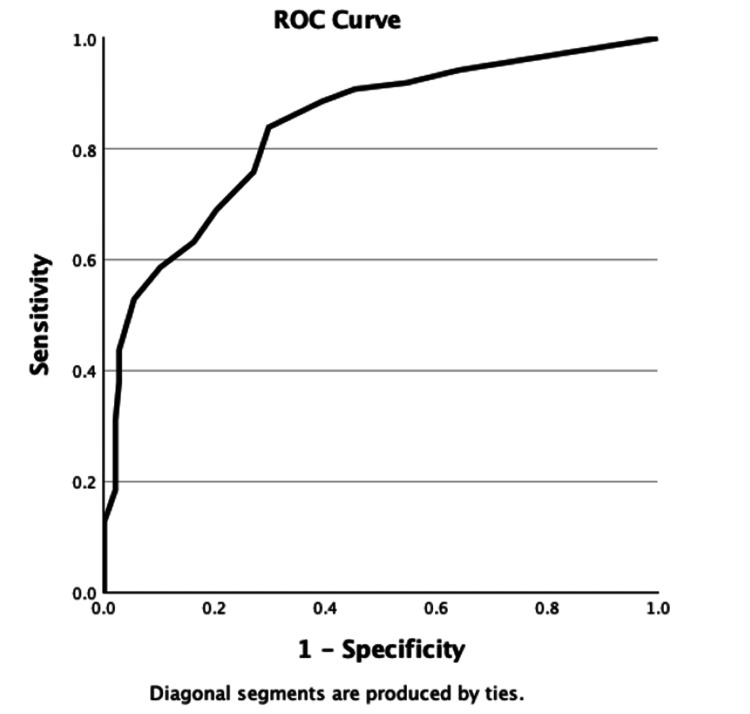
Receiver operating characteristic (ROC) curve of ABNAS mental slowing against PCFS grades, grade of at least 2 taken as true positives ABNAS: A-B Neuropsychological Assessment Schedule, PCFS: post-COVID functional status

## Appendices

Appendix A 

Appendix B 

Appendix C 
